# Metrnl/Meteorin-like/IL-41, a novel regulator of bone metabolism and disease activity in ankylosing spondylitis: based on multi-omics analysis

**DOI:** 10.3389/fimmu.2025.1595181

**Published:** 2025-07-23

**Authors:** Zhuoqi Li, Tao Sun, Min Zhao, Liping Xia, Hui Shen

**Affiliations:** ^1^ Department of Rheumatology and Immunology, The First Hospital of China Medical University, China Medical University, Shenyang, China; ^2^ National Clinical Research Center for Laboratory Medicine, Department of Laboratory Medicine, The First Hospital of China Medical University, Shenyang, China

**Keywords:** Metrnl, autoimmune disease, biomarker, multi-omics, bone metabolism

## Abstract

**Background:**

Ankylosing spondylitis (AS) is an autoimmune disease characterized by bone destruction and abnormal remodeling. Metrnl, a secreted protein involved in inflammation and immune regulation, has recently been linked to bone growth. This study aimed to evaluate serum Metrnl levels in AS patients and explore its bone regulatory mechanisms using cell models and multi-omics analyses.

**Methods:**

A total of 275 participants aged 16–60 years were included to measure serum Metrnl levels using Enzyme-Linked-Immunosorbent Assay (ELISA). Correlation and receiver operating characteristic (ROC) curve analyses assessed the diagnostic and predictive value of Metrnl. Mouse pre-osteoblastic MC3T3-E1 cells were treated with recombinant Metrnl (0/10/50 ng/mL) during 28-day osteogenic differentiation. RT-qPCR and alkaline phosphatase (ALP)/Alizarin Red S (ARS) staining was used to evaluate direct osteogenic differentiation effects. Transcriptomic and proteomic studies were conducted to further explore bone metabolism mechanisms. Finally, multi-omics integration analyses identified key pathways and targets.

**Results:**

Elevated serum Metrnl levels correlated directly with disease activity markers (CRP, ESR, IL-6) in AS-Active patients, but not in AS-Stable patients. ROC analysis validated Metrnl as a potential auxiliary diagnostic biomarker for high disease activity. *In vitro*, Metrnl suppressed ALP/OCN expression without altering overall osteogenic differentiation. Transcriptomic and proteomic analyses revealed Metrnl’s regulatory effects on osteogenic genes and proteins, emphasizing its role in bone and cartilage development. Bioinformatics highlighted Metrnl’s inhibition of endochondral ossification, delaying cartilage development and promoting osteoclast differentiation. Multi-omics integration identified Aspn and Sp7 as key targets in bone remodeling and resorption balance.

**Conclusions:**

Metrnl may serve as an additional diagnostic biomarker for AS and as an indicator for monitoring AS disease activity. Besides, Metrnl plays a critical role in regulating cartilage and bone metabolism and maintaining bone homeostasis, providing new insights for the future diagnosis and treatment of bone-related diseases.

## 1 Introduction

Ankylosing spondylitis (AS), or radiographic axial spondyloarthritis (axSpA), is a chronic autoinflammatory disease affecting the spine and sacroiliac joints, leading to abnormal bone remodeling and reduced mobility (1, 2). Although the pathogenesis is not fully understood, the progression of AS typically involves inflammation, fatty repair, and eventual new bone formation (3, 4). Recent studies has emphasized the importance of cytokine-mediated immune responses in the progression of AS, positioning cytokines as key targets for biomarkers and therapies (5–7). However, the early diagnosis of AS remains challenging, and current treatments often fail to prevent new bone formation. Therefore, identifying novel molecular biomarkers or therapeutic targets to inhibit bone formation and improve patient outcomes is essential.

Metrnl, also referred to as Meteorin-like, Meteorin-β, Subfatin, Cometin, and IL-41, is a novel secretory protein involved in inflammation, immunology, and metabolic regulation that is abundant in organs related to metabolism and barrier tissues (8). Numerous studies have demonstrated that Metrnl can reduce inflammation in adipose (9–11), muscle (12–14), skin (15), and lung tissues (16). Furthermore, its overexpression can alleviate inflammation and endoplasmic reticulum stress *in vitro* in injured cells, whereas its deficiency may promote inflammation development (17). Current research in inflammatory diseases indicates that circulating Metrnl levels are positively correlated with disease activity in conditions such as fulminant hepatitis (11), hyperuricemia (18), sepsis (19, 20), allergic asthma (21), and atopic dermatitis (15). However, the relationship between Metrnl serum levels and inflammatory cytokines appears inconsistent across different diseases, suggesting the need for larger studies to clarify the relationship between Metrnl and the network of inflammatory factors.

Given the immunomodulatory properties of Metrnl, research has explored its role in abnormal immune responses, particularly in autoimmune diseases. A recent study highlighted Metrnl’s role in the host immunity defense during the early phase of sepsis by promoting macrophage recruitment and balancing Treg/Th17 immune cell levels, although this protective effect was lost as patients’ conditions worsened or led to death (19). Additional research on various autoimmune diseases has found that circulating Metrnl levels correlate with disease activity, including in acute gout (22), myasthenia gravis (23), and Graves’ disease (24). Our previous work and another study have both indicated that serum Metrnl levels were elevated in rheumatoid arthritis (RA) patients and positively correlated with disease activity (25, 26). This finding is consistent with studies demonstrating Metrnl up-regulation in the synovial fluid of patients with psoriatic arthritis (PsA) and RA, but with decreased levels in the serum of patients with osteoarthritis (OA) (27). Despite these findings, the specific mechanisms of Metrnl in immune-mediated arthritis and other autoimmune diseases are still not well understood, necessitating further research.

Recent research suggested that Metrnl is involved in bone growth, development, remodeling, and related diseases (16, 28). After constructing a human osteoblast cDNA library to identify genes closely associated with the transcription factor AP-1, Metrnl was recognized as the sole candidate gene, suggesting a genomic link between Metrnl and osteoblast function (29). Additionally, a strong positive correlation has been observed between circulating Metrnl levels and osteogenic molecules in metabolic diseases (30), though its precise mechanisms remain unclear. Moreover, Metrnl promotes osteoblast differentiation and fracture healing *in vitro*, with high expression observed in fracture callus (31). Sequencing data highlight Metrnl as a top transcript in osteoblasts, and its knockout reduces bone marrow stromal cell osteogenic capacity (31). Conversely, overexpression of Metrnl may inhibit osteoblast mineralization by downregulating OCN and AP-1, maintaining osteoblasts in matrix formation rather than mineralization (29). Thus, further comprehensive studies on Metrnl’s role in osteoblasts are warranted.

Considering that AS is characterized by axial inflammation, early bone destruction, and late ectopic ossification characteristic of AS, the roles of Metrnl in bone formation and resorption remain unclear. Therefore, this study aimed to evaluate serum Metrnl levels in AS patients and explore its role in regulating bone metabolism using *in vitro* cell models and multi-omics analyses.

## 2 Methods

### 2.1 Patients and controls recruitment

This study enrolled 275 participants (age range: 16–60 years), including 150 patients with AS, 20 with PsA, and 105 healthy controls (HCs). All participants were recruited from the First Hospital of China Medical University (Shenyang, China) between December 2021 and September 2023. All patients were treatment-naive. HCs were healthy voluntary blood donors without any diagnosed diseases, who presented for routine physical examinations during the same period. Demographics and clinical characteristics of AS, PsA patients, and HCs are shown in **Supplementary Table S1**.

AS patients met the 2009 Assessment of SpondyloArthritis International Society (ASAS) classification criteria for axSpA (32). Disease activity in AS was assessed using the Bath Ankylosing Spondylitis Disease Activity Index (BASDAI) (33). Patients were stratified into two groups based on BASDAI scores: AS-Stable group (n=74, BASDAI ≤ 4) and AS-Active group (n=76, BASDAI > 4) PsA patients met the 2009 Group for Research and Assessment of Psoriasis and Psoriatic Arthritis (GRAPPA) diagnostic criteria (34). The specific exclusion/inclusion criteria are listed in the **Supplementary Materials**.

### 2.2 Ethical considerations

This study was conducted following the Declaration of Helsinki. All participants provided informed written consent for all research procedures. The research protocol received approval from the Ethics Committee on Human Experimentation at the First Hospital of China Medical University (license number: AF-SOP-07-1. 1-01).

### 2.3 Clinical and laboratory indicators

Clinical characteristics and laboratory data were extracted from the electronic medical records. The following parameters were analyzed: disease activity markers (BASDAI, CRP, ESR), immunological indicators (HLA-B27, C3, C4, IgG, IgA, IgM, UA), blood biochemistry (AST, ALT, ALP, GGT, BUN, Cr, Cys-C, TG, TC, HDL-C, LDL-C), and routine blood parameters (WBC, LY, RBC, HGB, PLT). Serum inflammatory cytokines (IL-6, IL-17, TNF-α, IFN-γ) were quantified using ELISA.

### 2.4 Sample collection and preparation

Venous blood samples (2 mL) were collected from fasting participants. Samples were centrifuged at 2500 rpm for 15 minutes to separate serum, which was stored at -20°C for short-term (≤1 month) or -80°C for long-term use. The supernatant was used for Metrnl ELISA assays.

### 2.5 Cell culture and osteogenic induction

Mouse calvarial osteoprogenitor cells (MC3T3-E1 subclone 14) were used as the *in vitro* model system. Cells were cultured in basic medium (BM: 90% MEMα, 10% FBS, 1% P/S), refreshed every 2–3 days. At 95% confluency, cells were passaged at a 1:2 ratio using trypsin-EDTA. Six-well plates were pre-coated with 0.5% gelatin solution (1 mL/well) overnight at 4°C, air-dried, and used for experiments. Osteogenic induction medium was prepared by supplementing BM with 50 μg/mL ascorbic acid, 10 mM β-glycerophosphate, and 10 nM dexamethasone. The medium was introduced gradually over 48h using a stepwise concentration increase (25%, 50%, 100%).

### 2.6 Drug intervention

Recombinant human METRNL protein (Abcam, ab267871) was dissolved in sterile water to prepare a 100 μg/mL stock solution. The stock solution was diluted with osteogenic induction medium to achieve final working concentrations of 10 ng/mL and 50 ng/mL. Cells (2nd–6th passage, 70% confluency) were seeded at 1×10^5^ cells/mL (2×10^5^ cells/well) in BM. After stabilization, cells were treated with Metrnl (10 ng/mL or 50 ng/mL) for 28 days. Medium was fully replaced every 2 days for the first 10 days and partially (70%) every 4 days thereafter, without rinsing after day 10 to maintain cell adherence.

### 2.7 ELISA

Serum Metrnl levels were measured using an ELISA kit (R&D Systems). Capture antibodies were coated onto wells overnight at 4°C. Afterward, samples and standards were incubated After washing, detection antibody was added for 2 hours. Then, HRP substrate (30 min) and TMB substrate (20 min) were added in the dark. The reaction was stopped with stop solution, and absorbance was measured at 450 nm using a microplate reader (Awareness 4700, USA). Data were exported to Excel for analysis.

### 2.8 Alkaline phosphatase staining and Alizarin Red S staining

ALP staining was performed on Days 7 and 9, while ARS staining was conducted on Days 21 and 28. For ALP staining, cells were fixed with 4% paraformaldehyde for 15 minutes after two washes with PBS at room temperature. The BCIP/NBT staining solution was prepared by combining 6 mL of ALP solution with 20 μL BCIP and 60 μL NBT), then added to the wells. Cells were incubated in the dark for 2–6 hours until stable coloration developed. For the ARS staining, cells were similarly fixed with paraformaldehyde for 30 minutes, and then stained with Alizarin Red solution for 10 minutes in darkness. Upon achieving stable coloration, both ALP and ARS staining solution was discarded and rinsed with double-distilled water (ddH2O) twice. Cell imaging was performed on a Cytation5 Cell Imaging Microplate Detection System (BioTek, USA) with 4x and 20x objectives. The original images were subsequently exported in Tiff format.

### 2.9 RT-qPCR

Total RNA was extracted using TransZol Up, quantified with a NanoDrop spectrophotometer, and stored at −80°C. To remove genomic DNA, RNA samples were treated with gDNA Digester Mix, and cDNA was synthesized using Super Mix enzyme in a thermal cycler under a standard program. The cDNA was diluted and stored at low temperature. A qPCR mixture of 10 μL containing cDNA, primers, DEPC-treated water, and SYBR Green qPCR Mix was prepared. qPCR was performed with 40 cycles at 95°C (denaturation) and 60°C (annealing/extension). The target gene expression including ALP, bone sialoprotein (BSP), osteocalcin (OCN), osteopontin (OPN), and Runt-related transcription factor 2 (Runx2) was normalized to β-Actin as the reference gene (primer sequences provided in **Supplementary Table S2**).

### 2.10 RNA-seq

Total RNA was collected on Day 28 from MC3T3-E1 cells undergoing osteogenic differentiation and sent to BGI Genomics Co., Ltd. (Shenzhen, China) for eukaryotic transcriptome sequencing. The experimental group was treated with 50 ng/mL recombinant METRNL protein, while the control group received a blank treatment. Each group consisted of three biological replicates. The sequencing was performed on a BGISEQ platform, using PE150 technology, and the raw data in FASTQ format will be provided.

### 2.11 Quantitative proteomics

Total cells were collected from MC3T3-E1 cells were collected after 28 days of osteogenic differentiation and sent to Hangzhou Jingjie Biotechnology Co., Ltd. (Hangzhou, China) for quantitative proteomics analysis. The experiment group was treated with 50 ng/mL recombinant METRNL protein, while the control group received a blank treatment. Each group included three biological replicates.

### 2.12 Bioinformatics analysis

Proteomics raw data were preprocessed by searching protein databases, performing quality control by peptide segments, quantifying proteins, and assessing repeatability. Functional annotation and Gene Set Enrichment Analysis (GSEA) were performed for the identified proteins, followed by differential analysis to determine differentially expressed proteins (DEPs). Functional classification, functional enrichment analysis, and clustering analysis were then performed on the DEPs. RNA-seq raw data was analyzed to determine gene expression levels for each sample. GSEA was performed on the identified genes, followed by differential analysis to identify differentially expressed genes (DEGs). Annotation categorization and enrichment analysis were conducted on the DEGs. Finally, we conducted an integrated analysis of transcriptomics and proteomics data, including correlation analysis, differential correlation analysis, and enrichment correlation analysis. Additionally, we presented intersections and correlations between DEPs and DEGs by plotting a nine-quadrant diagram, and correlation scatter plots, to identify potential targets.

### 2.13 Statistical analysis

Data were analyzed using SPSS software (IBM Corporation, Armonk, NY, USA), and figures were created with OriginPro software (OriginLab Corporation, Northampton, MA, USA). Continuous numerical variables were evaluated for normal distribution using the Shapiro-Wilk test. Data adhering to normality were described using mean and standard deviation, and analyzed with two independent sample t-tests to compare statistical differences. Data not following a normal distribution were described using median and interquartile range (IQR). Group comparisons were conducted using one-way ANOVA with a significance level set at p < 0.05. Categorical data were analyzed using the Mann-Whitney U test and Fisher’s exact test to assess group differences and associations. Binary logistic regression was utilized to evaluate the impact of combinations of indicators on predicting disease activity. Pearson correlation analysis was employed to assess correlations between parameters. The receiver operating characteristic (ROC) curve and its area under the curve (AUC) were analyzed to evaluate the sensitivity and specificity of biomarkers for predicting higher disease activity.

## 3 Results

### 3.1 Serum Metrnl levels elevated in AS-active period patients

**Figure 1:**
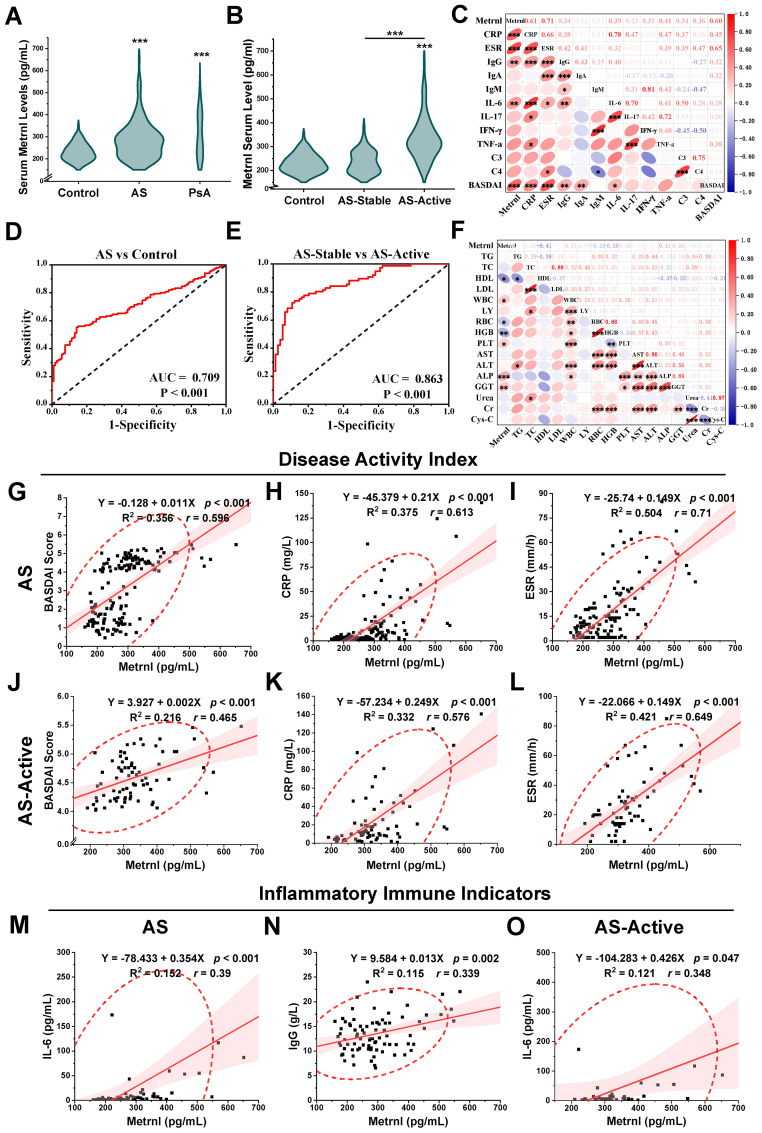
Serum Metrnl levels and correlation analyses among clinical features. **(A)** Serum Metrnl levels in AS (n=150), PsA (n=20), and Controls (n=105). Data were presented as violin plots with median and IQR. **(B)** Serum Metrnl levels in AS-Active (n=74), AS-Stable (n=74), and Controls (n=105). AS-Active, AS patients in active period; AS-Stable, AS patients in stable period. **(C)** Correlation heat map among inflammatory immune indicators of AS patients. **(D)** ROC curves for prediction of AS disease activity; sensitivity: 72.4%, specificity: 89.2%. **(E)** ROC curves for prediction of AS from healthy controls; sensitivity: 55.3%, specificity: 85.8%. **(F)** Correlation heat map among biochemical index of AS patients. **(G)** Linear correlation map between Metrnl and BASDAI score in AS. **(H)** Linear correlation map between Metrnl and CRP in AS. **(I)** Linear correlation map between Metrnl and ESR in AS. **(J)** Linear correlation map between Metrnl and CRP in AS-Active. **(L)** Linear correlation map between Metrnl and ESR in AS-Active. **(M)** Linear correlation map between Metrnl and IL-6 in AS. **(N)** Linear correlation map between Metrnl and IgG in AS. **(O)** Linear correlation map between Metrnl and IL-6 in AS-Active. *p < 0.05, **p < 0.01, ***p < 0.001.

To determine whether serum Metrnl levels vary in patients with AS, we enrolled 150 AS patients, 20 PsA patients, and 105 HCs aged 16–60 years. Metrnl concentrations were significantly higher in both AS (277.57 pg/mL, IQR: 218.94 - 323.98) and PsA patients (286.59 pg/mL, IQR: 219.15 - 403.61) compared to HCs (223.30 pg/mL, IQR: 200.69 - 258.08, *p* < 0.001) (**Figure 1A**; **Supplementary Table S3**). Specifically, Metrnl levels were markedly elevated in AS-Active period patients (**Figure 1B**; 317.64 pg/mL, IQR: 283.46 - 369.80) compared to AS-Stable periods (228.44 pg/mL, IQR: 193.59 - 273.69, *p* < 0.001). Although Metrnl levels appeared slightly higher in AS-Stable patients than in healthy controls, the difference was not statistically significant (*p* = 0.553).

### 3.2 Serum Metrnl levels positively correlated with disease activity in AS patients

Correlation analyses were performed between serum Metrnl levels and clinical indicators in the AS group (AS-Active and AS-Stable) (**Table 1**). Regarding disease activity indicators, serum Metrnl levels in AS patients showed a positive correlation with the BASDAI scores (**Figure 1G**; *r* = 0.596, *p* < 0.001), CRP (**Figure 1H**; *r* = 0.613, *p* < 0.001), and ESR (**Figure 1I**; *r* = 0.71, *p* < 0.001). This correlation was especially pronounced in the AS-Active group, with BASDAI scores (**Figure 1J**; *r* = 0.465, *p* < 0.001), CRP (**Figure 1K**; *r* = 0.576, *p* < 0.001), and ESR (**Figure 1L**; *r* = 0.649, *p* < 0.001). However, no correlation was observed in the AS-Stable group. Regarding inflammatory markers, serum Metrnl levels in AS patients were only positively correlated with IL-6 (**Figure 1M**; *r* = 0.39, *p* < 0.001), with no significant correlation with IL-17, TNF-α, and IFN-γ. In the AS-Active group, a similar positive correlation was found between Metrnl and IL-6 (**Figure 1O**; *r* = 0.348, *p* = 0.047), but no correlation was noted with other inflammatory markers in the AS-Stable group. Regarding immune indicators, serum Metrnl levels in AS patients correlated positively only with IgG (**Figure 1N**; *r* = 0.339, *p* = 0.002), but not with IgM, IgA, C3, and C4. No significant correlations were observed in either the AS-Active or AS-Stable groups. These relationships are further visualized in heat maps (**Figures 1C, F**).

**Table 1:** Correlations between Metrnl and clinical features in patients with AS.

Variables	AS		AS-Active		AS-Stable	
	*r*	*p*	*r*	*p*	*r*	*p*
Age	0.048	0.556	-0.028	0.81	-0.227	0.052
BASDAI score	0.596	**<0.001**	0.465	**<0.001**	0.056	0.637
CRP	0.613	**<0.001**	0.576	**<0.001**	0.049	0.684
ESR	0.71	**<0.001**	0.649	**<0.001**	-0.156	0.198
IL-6	0.390	**<0.001**	0.348	**0.047**	0.004	0.981
IL-17	0.230	0.328	0.171	0.511	**/**	**/**
TNF-α	-0.027	0.91	0.305	0.234	**/**	**/**
IFN-γ	0.307	0.188	0.269	0.296	**/**	**/**
IgG	0.339	**0.002**	0.239	0.119	0.078	0.631
IgA	0.124	0.262	-0.1	0.517	-0.108	0.506
IgM	0.106	0.338	0.132	0.392	0.008	0.961
C3	0.34	0.104	0.4	0.072	0.18	0.885
C4	0.356	0.087	0.351	0.119	0.942	0.218
WBC	0.212	**0.016**	0.205	0.111	0.016	0.898
LY	0.131	0.139	0.117	0.365	0.004	0.975
RBC	-0.218	**0.014**	-0.347	**0.006**	0.214	0.085
HGB	-0.284	**<0.001**	-0.339	**0.007**	0.249	**0.043**
PLT	0.186	**0.036**	0.227	0.076	-0.267	**0.031**
TG	-0.011	0.955	-0.078	0.716	0.038	0.962
TC	-0.083	0.675	-0.045	0.835	-0.055	0.945
HDL	-0.41	**0.03**	-0.282	0.182	-0.876	0.124
LDL	0.032	0.871	-0.014	0.948	0.706	0.294
AST	-0.044	0.629	-0.045	0.729	0.021	0.873
ALT	0.051	0.577	0.089	0.49	-0.031	0.816
ALP	0.313	**<0.001**	0.214	0.091	0.082	0.534
GGT	0.24	**0.008**	0.161	0.208	0.052	0.694
Urea	-0.069	0.484	-0.045	0.734	-0.026	0.866
Cr	-0.065	0.506	-0.135	0.306	-0.005	0.975
Cys-C	-0.008	0.936	0.186	0.162	-0.036	0.819

*BASDAI, Bath Ankylosing Spondylitis Disease Activity Index; CRP, C-reactive protein; ESR, erythrocyte sedimentation rate; IL-6, interleukin-6; IL-17, interleukin-17A; TNF-α, tumor necrosis factor-α; IFN-γ, interferon-γ; IgG, immunoglobin G; IgA, immunoglobin A; IgM, immunoglobin M; C3, complement 3; C4, complement 4; WBC, white blood cell count; LY, lymphocyte; RBC, red blood cell count; HGB, hemoglobin; PLT, platelet count; TG, triglyceride; TC, total cholesterol; HDL, high-density lipoprotein; LDL, low-density lipoprotein; AST, aspartate transaminase; ALT, alanine aminotransferase; ALP, alkaline phosphatase; GGT, γ-glutamyl transpeptidase; Cr, creatinine; Cys-C, cysteine-C.

The bold values denote statistical significance at the P < 0.05 level.

### 3.3 Metrnl could monitor AS disease activity combined with traditional indicators

ROC curve analyses were performed to assess the diagnostic and predictive value of serum Metrnl levels in AS. The optimal cutoff value of Metrnl to distinguish AS from healthy controls was 270.46 pg/mL, with 85.8% specificity and 55.3% sensitivity (**Figure 1D**). The AUC was 0.709 (*p* < 0.001, 95%CI: 0.646 - 0.771). A separate univariate ROC curve analysis evaluated the potential of Metrnl in monitoring high disease activity in AS using comparisons between AS-Stable and AS-Active groups. The optimal cutoff value of Metrnl for distinguishing the AS-Active period was 290.49 pg/mL, with a specificity of 89.2% and sensitivity of 72.4% (**Figure 1E**). The AUC was 0.863 (*p* < 0.001, 95%CI: 0.806 - 0.921). Further analysis of ROC curves for other disease activity indicators, including CRP, ESR, IL-6, C3, C4, IgG, IgA, and IgM, was conducted to compare their diagnostic values against serum Metrnl levels (**Table 2**). The predictive value of CRP, ESR, and IL-6 was higher than that of serum Metrnl levels, although not statistically significant. Combining Metrnl with CRP, ESR, and IL-6 enhanced the accuracy of monitoring high disease activity.

**Table 2:** Airwise comparisons of ROC curves between AS-Stable and AS-Active groups.

Variables	AS-Active vs. AS-Stable		
	AUC	95% CI	P value
Metrnl	0.863	0.806 - 0.921	1^Ref^
CRP	0.910	0.861 – 0.959	0.235
ESR	0.911	0.858 – 0.965	0.427
IL-6	0.944	0.888 – 0.999	0.322
IgG	0.674	0.559 – 0.788	**0.012**
IgA	0.686	0.572 – 0.800	**0.034**
IgM	0.453	0.327 – 0.578	**<0.001**
C3	0.460	0.236 – 0.684	**0.001**
C4	0.492	0.158 – 0.827	**0.008**
Combine-Metrnl-CRP	0.941	0.906 – 0.976	**0.001**
Combine-Metrnl-ESR	0.949	0.913 – 0.986	**0.008**
Combine-Metrnl-IL-6	0.971	0.941 – 1.002	**0.015**
Combine-Metrnl-IgG	0.867	0.791 – 0.943	0.361
Combine-Metrnl-IgA	0.898	0.834 – 0.961	0.102
Combine-Metrnl-IgM	0.848	0.767 – 0.928	0.770
Combine-Metrnl-C3	0.921	0.785 – 1.057	0.729
Combine-Metrnl-C4	0.952	0.865 – 1.039	0.340

*Ref, reference; 95%CI, 95% confidence interval for difference.

The bold values denote statistical significance at the P < 0.05 level.

### 3.4 Metrnl exerts no overall effect on osteogenic differentiation of MC3T3-E1 cells

**Figure 2:**
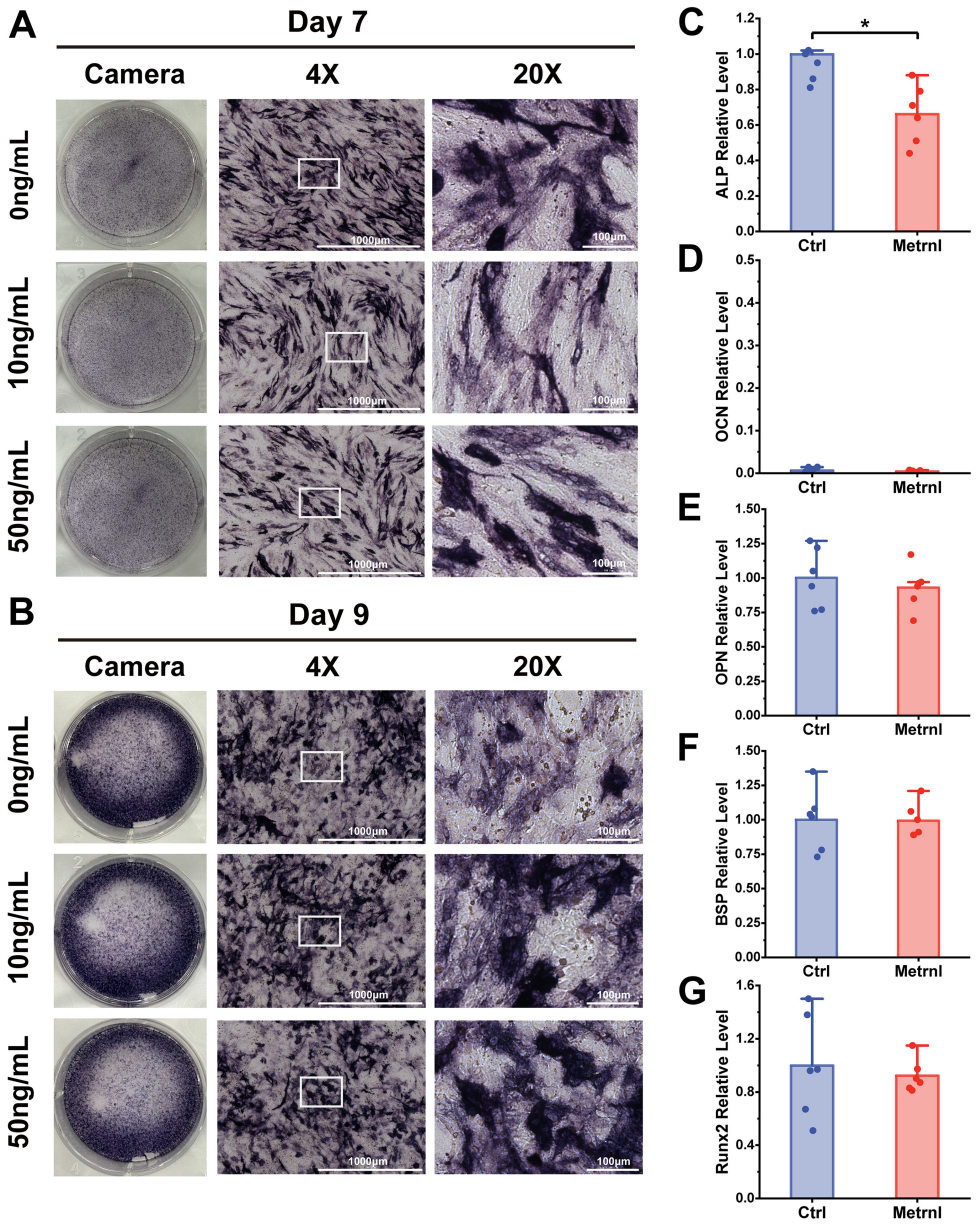
Effects of Metrnl intervention on early osteogenic differentiation. **(A, B)** ALP staining on 7d and 9d to assess early osteogenic differentiation and functional maturation. The image ruler was 1000μm upon 4X microscope and 100μm upon 20X microscope. **(A)** ALP staining on 7d. **(B)** ALP staining on 9d. **(C–E)** Metrnl’s effects on early osteogenic differentiation-related gene expression. The experiment group was treated with 50ng/mL Metrnl. Total RNA from all groups was extracted on 7d. **(C)** mRNA relative expression levels of ALP on 7d osteogenic differentiation. **(D)** mRNA relative expression levels of OCN on Day7 osteogenic differentiation. **(E)** mRNA relative expression levels of OPN on Day7 osteogenic differentiation. **(F)** mRNA relative expression levels of BSP on Day7 osteogenic differentiation. **(G)** mRNA relative expression levels of Runx2 on 7d osteogenic differentiation. *p < 0.05.

**Figure 3:**
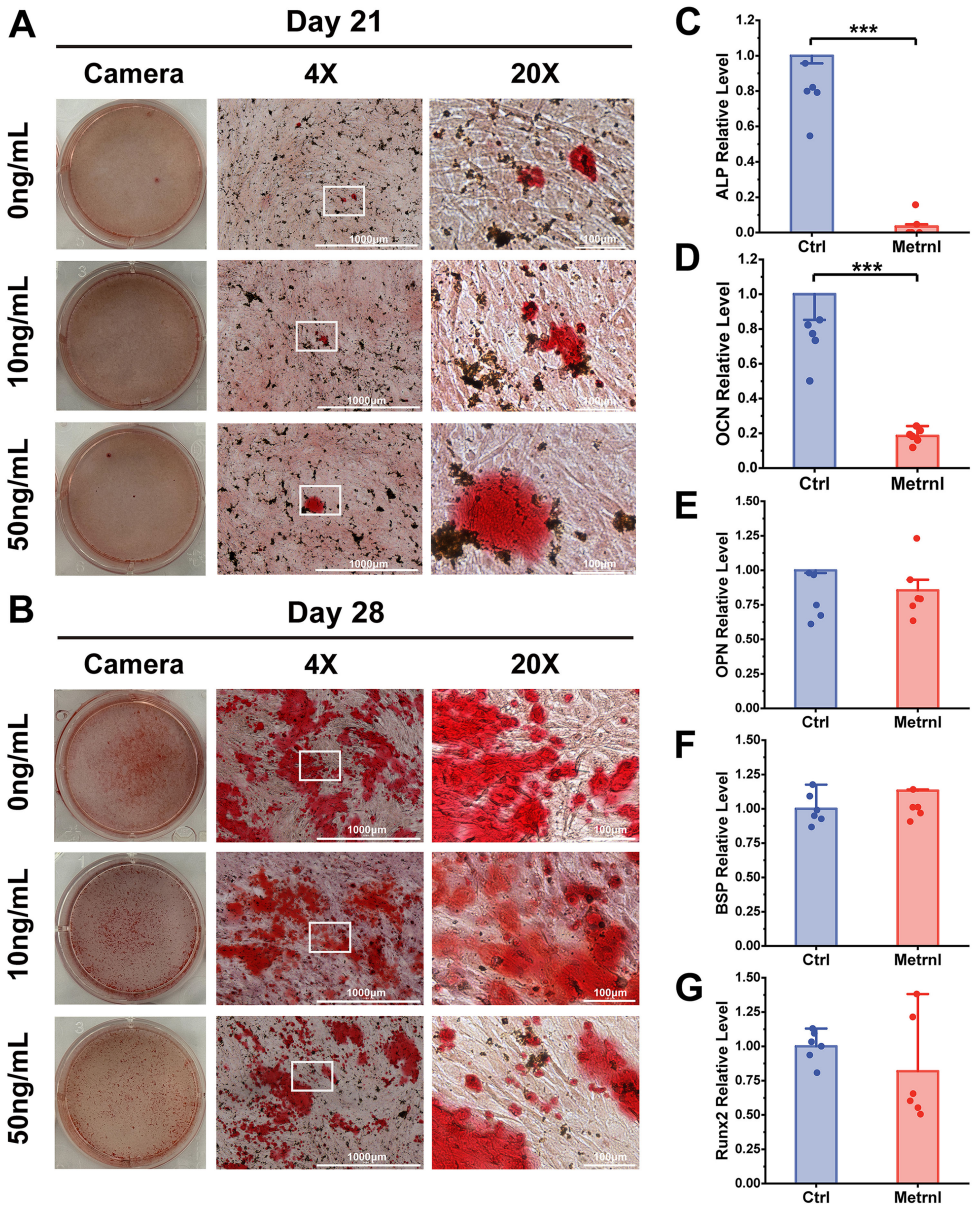
Effects of Metrnl intervention on late osteogenic differentiation stage. **(A, B)** ARS staining on 21d and 28d to assess calcified nodule formation. **(A)** ARS staining on 21d. **(B)** ARS staining on 28d. **(C–E)** Metrnl’s effects on late-stage osteogenic differentiation-related gene expression. The experiment group was treated with 50ng/mL Metrnl. Total RNA from all groups was extracted on 21d. **(C)** mRNA relative expression levels of ALP on 21d osteogenic differentiation. **(D)** mRNA relative expression levels of OCN on 21d osteogenic differentiation. **(E)** mRNA relative expression levels of OPN on 21d osteogenic differentiation. **(F)** mRNA relative expression levels of BSP on 21d osteogenic differentiation. **(G)** mRNA relative expression levels of Runx2 on 21d osteogenic differentiation. *p < 0.05.

To investigate the overall effects of Metrnl on osteogenic differentiation, the mouse embryonic pre-osteoblast MC3T3-E1 cell line was employed as an *in vitro* model. Osteogenic induction was initiated in MC3T3-E1 cells, with Metrnl added at concentrations of 0 ng/mL, 10 ng/mL, and 50 ng/mL for continuous induction over 28 days. ALP staining was performed on Days 7 and 9 to assess early osteogenic differentiation markers, while ARS staining was conducted on Days 21 and 28 to evaluate calcified nodule formation. Early osteogenic induction progressed over time, with all groups showing an increase in the amount and intensity of blue-purple precipitate from Day 7 to Day 9 (**Figures 2A, B**). However, no significant differences in ALP staining were observed between the Metrnl-treated groups and the control group. In the late-stage ARS staining, only a few red precipitates were observed under the microscope on Day 21 across all groups, with no differences between the experimental and control groups (**Figures 3A, B**). On Day 28, large patches of orange-red precipitates were visible to the naked eye within the plates, with a slight trend towards a reduced area of precipitates in the Metrnl-treated groups compared to the control, but the differences were minor.

### 3.5 Metrnl inhibits osteogenic differentiation-related genes expression

To further investigate Metrnl’s effects on gene expression during osteogenic differentiation, total RNA was collected on Days 7 and 21 at various stages of osteogenic induction (experimental group: 50 ng/mL, control group: 0 ng/mL). RT-qPCR was used to detect the mRNA expression levels of osteogenic-related genes ALP, OCN, Runx2, BSP, and Col1a, monitoring the changes in osteogenic markers at different differentiation stages. ALP expression, an early osteogenic marker, was significantly reduced in the Metrnl-treated group throughout the differentiation process (**Figures 2C**, **3C**). The matrix mineralization marker OCN began to be expressed in the later stages of osteogenesis, with significantly lower expression upon Metrnl stimulation compared to the control group (**Figures 2D**, **3D**). However, no significant differences were noted in the expression levels of OPN (**Figures 2E**, **3E**), BSP (**Figures 2F**, **3F**), and the osteogenic transcription factor Runx2 (**Figures 2G**, **3G**), and between the experimental and control groups.

### 3.6 Transcriptomic analysis of Metrnl stimulation on osteogenic differentiation

#### 3.6.1 Metrnl modulates osteogenic gene expressions

To elucidate the mechanisms underlying Metrnl’s effects on osteogenic differentiation, RNA was extracted on Day 28 for RNA sequencing. A total of 15,391 genes were detected in both groups. The Metrnl-treated group uniquely expressed 830 genes, including 13 DEGs: *Cxcl5, Trpm2, Cxcl1, Serpina3k, Saa2, Tnfrsf18, Vnn3, Pglyrp2, Saa1, Rspo4, H2ac13, Gm21677*, and *Tnfsf14*. In contrast, the control group had 791 unique genes but no DEGs. From the gene expression matrix, 402 DEGs were identified (|log2FC| ≥ 1.5; adj.P.Value< 0.05), with 271 up-regulated and 131 down-regulated genes. Key up-regulated genes included *C3, Lcn2, Tlr2, Cxcl12, Hp, Nfkbiz, Tnfaip3, Ccl2, Saa3*, and *Lrig1*. Down-regulated genes included *Aspn, Omd, Npnt, Snta1, Arl4d, Rflnb, Plaat3, Spon2, Phex*, and *Msrb1* (**Supplementary Figures S2A, B**). Notably, *Aspn* and *Rflnb* are critical for cartilage homeostasis, while *Omd* and *Npnt* function in bone mineralization, suggesting that Metrnl alters bone-related gene expression during osteoblast differentiation.

#### 3.6.2 GSEA highlights Metrnl’s role in bone formation and osteoclast differentiation

**Figure 4:**
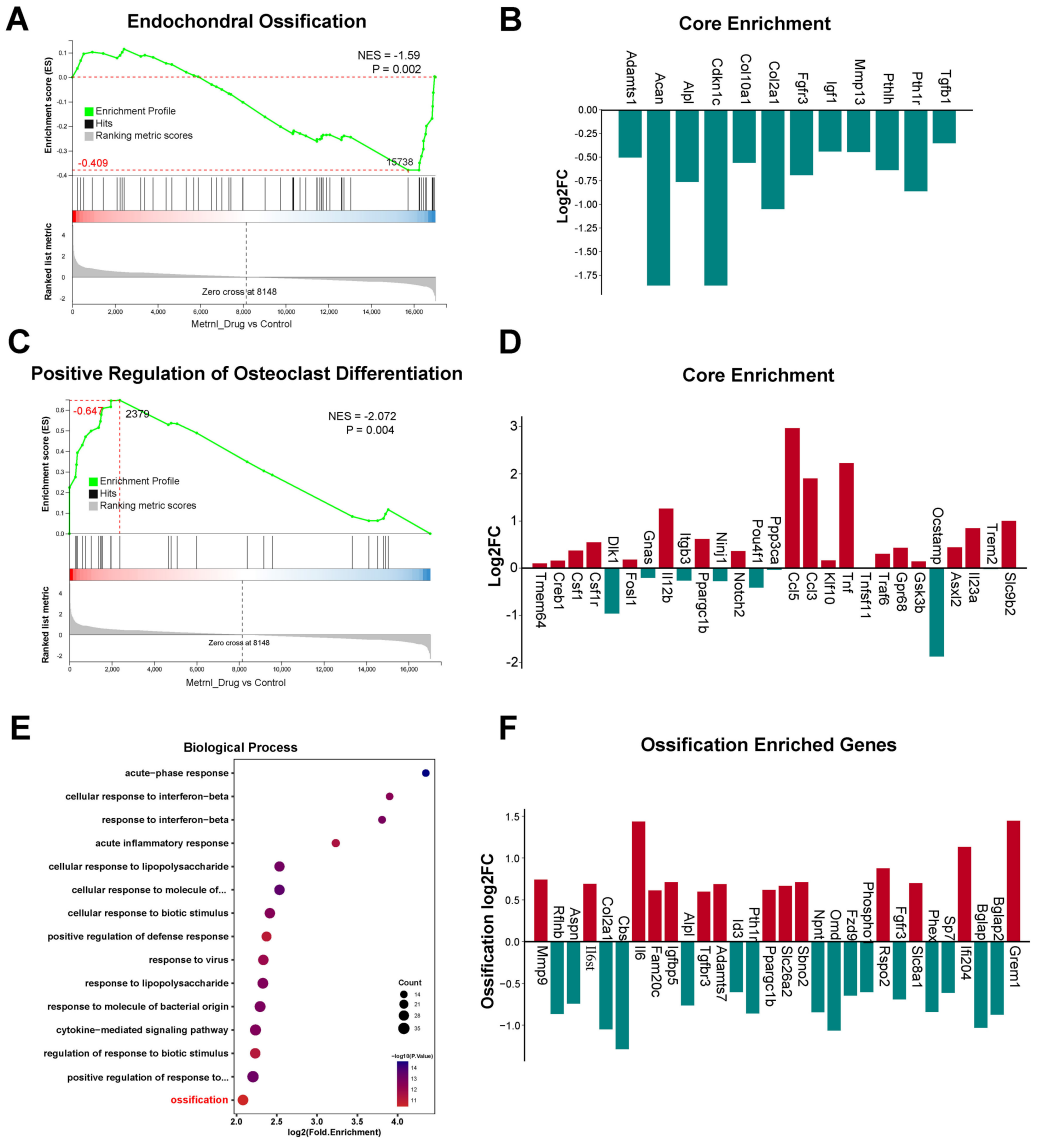
Transcriptomic analysis of Metrnl stimulation on MCETE-E1 cells osteogenic differentiation. Total RNA was extracted on 28d for RNA sequencing. The experiment group was treated with 50ng/mL Metrnl. **(A, B)** GSEA analyses the gene sets enriched in the pathway of Endochondral Ossification. **(A)** GSEA map of negatively regulated Endochondral Ossification. NES, Normalized Enrichment Score. **(B)** Bar graph of the core gene set downregulated in the pathway of Endochondral Ossification. **(C, D)** Enrichment maps of gene set enriched in Positive Regulation of Osteoclast Differentiation by GSEA analysis. **(C)** GSEA map of positively regulated Positive Regulation of Osteoclast Differentiation. **(D)** Bar graph of the core gene set upregulated in the pathway of Positive Regulation of Osteoclast Differentiation. **(E, F)** Enrichment maps of DEGs enriched in the Ossification pathway in the Biological Process (BP) category of GO database. **(E)** Bubble plots of DEGs enriched in the BP category of the GO database. **(F)** Bar plots of DEGs enriched in the Ossification pathway.

GSEA revealed Metrnl’s dual regulatory role in bone metabolism. Based on the WikiPathways database, GSEA demonstrated a significant decrease of Endochondral Ossification pathway genes (p-value = 0.002) after Metrnl stimulation (**Figure 4A**). The affected pathway contained 61 genes, with 32 showing downregulation including 11 core genes (**Figure 4B**), indicating impaired cartilage-to-bone conversion during osteogenesis. Conversely, upregulation of Positive Regulation of Osteoclast Differentiation pathway (p-value = 0.004) was enriched, with 27 up-regulated core genes identified (**Figures 4C, D**), suggesting Metrnl enhanced osteoclast differentiation through microenvironment modulation.

**Figure 5:**
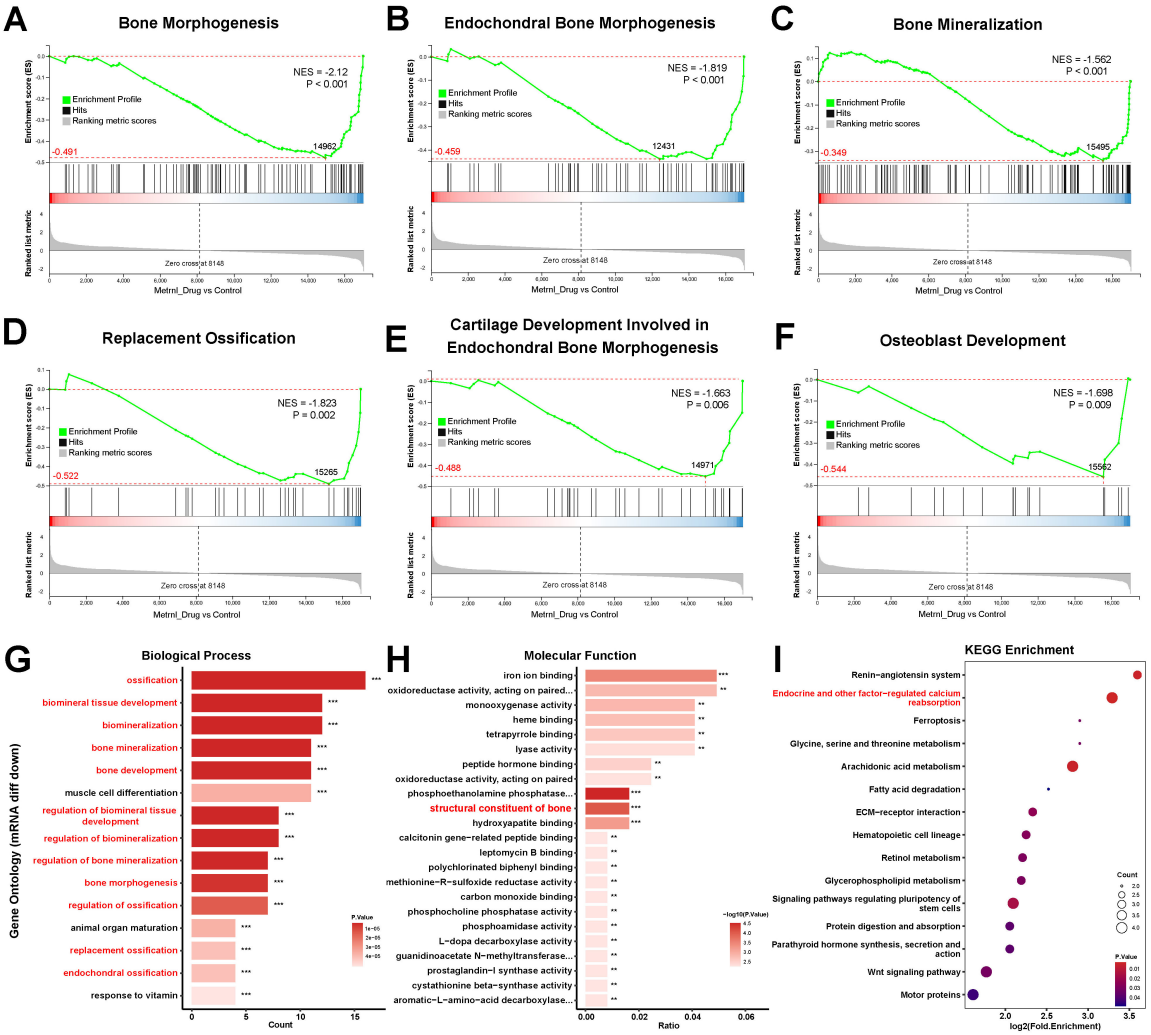
Transcriptomic analysis of Metrnl stimulation on MCETE-E1 cells osteogenic differentiation. **(A–F)** GSEA analyses gene sets associated with the skeletal system. **(A)** GSEA map of negatively regulated Bone Morphogenesis. **(B)** GSEA map of negatively regulated Endochondral Bone Morphogenesis. **(C)** GSEA map of positively regulated Bone Mineralization. **(D)** GSEA map of negatively regulated Replacement Ossification. **(E)** GSEA map of negatively regulated Cartilage Development Involved in Endochondral Bone Morphogenesis. **(F)** GSEA map of negatively regulated Osteoblast Development. **(G)** Bar plot of down-regulated DEGs associated with the skeletal system enriched in the biological process category of GO database. **(H)** Bar plot of down-regulated DEGs associated with the skeletal system enriched in the molecular function category of GO database. **(I)** Bubble plot of down-regulated DEGs pathway enrichment in KEGG database. **p < 0.01, ***p < 0.001.

Based on the Gene Ontology (GO) database, GSEA further identified multiple suppressed biological processes in bone development, remodeling, and replacement (NES<0; all p-value < 0.01). These pathways include Bone Morphogenesis (p-value < 0.001), Endochondral Bone Morphogenesis (p-value < 0.001), Bone Mineralization (p-value < 0.001), Replacement Ossification (p-value = 0.002), Cartilage Development Involved in Endochondral Bone Morphogenesis (p-value = 0.006), and Osteoblast Development (p-value = 0.009) (**Figures 5A–F**). These coordinated downregulations imply that Metrnl’s inhibitory role in bone formation and matrix mineralization, particularly delaying endochondral ossification through cartilage metabolism regulation. Concurrently, its promotion of osteoclast differentiation suggests a regulatory balance favoring bone resorption over formation.

#### 3.6.3 KEGG and GO enrichment analyses highlight Metrnl’s regulatory role in bone development

KEGG and GO enrichment analyses revealed Metrnl’s significant regulatory effects on skeletal development and ossification. GO analysis highlighted significant enrichment of DEGs in the Ossification pathway (biological process category, p-value < 0.05), with 30 DEGs identified (**Figures 4E, F**). Further analysis of 97 ossification-related genes revealed 20 up- and 20 down-regulated DEGs (p-value < 0.05), suggesting Metrnl modulates transcriptional programs critical for skeletal development and fracture repair.

Down-regulated DEGs exhibited significant enrichment in bone and cartilage regulatory pathways. Among the top 15 enriched GO terms (p-value < 0.01), 12 terms were directly governed in bone mineralization and ossification processes, including Endochondral Ossification, Bone Morphogenesis, and Biomineralization regulations (**Figure 5G**). Notably, pathways related to skeletal system maturation, such as osteoblast differentiation and cartilage development), were significantly suppressed. This suggests that Metrnl may decelerate osteochondral lineage progression during osteogenic induction. Molecular function analysis further revealed reduced expression of bone structural constituents, particularly Bglap and Bglap2 (**Figure 5H**), indicating that Metrnl affects bone matrix integrity.

KEGG pathway analysis found that downregulated DEGs prominently enriched in endocrine-regulated calcium reabsorption (p-value = 0.0007). Key mediators, including Pth1r and Klk family proteases (Klk1b21/24/27), showed marked suppression (**Figure 5I**). This suggests that Metrnl disrupts calcium homeostasis through transcriptional inhibition of these regulators.

Overall, integrating all transcriptome analysis results suggested that Metrnl influences bone formation and mineralization processes, potentially impeding the transformation from cartilage to bone during osteogenic differentiation. Additionally, several genes involved in bone growth and tissue development showed significant expression changes due to Metrnl stimulation.

### 3.7 Proteomic analysis of Metrnl stimulation on osteogenic differentiation

#### 3.7.1 Proteomic analysis reveals Metrnl’s regulatory effects on osteoblast differentiation

Quantitative proteomic analysis conducted on Day 28 identified 80 DEPs (|log2FC| ≥ 1.5, P. Value < 0.05), with 43 up-regulated and 37 down-regulated (**Supplementary Figures S2C, D**). Key up-regulated proteins included *C3, Cp, Ifitm3, Relb, Tmem176b, Cd14, C1ra, Tlr2, Mt2*, and *Ifit1*, while down-regulated proteins included *Adamts1, Rpf2, Adcy3, Rps10, Rab24, Sp7, Cfap58, Hba, Macroh2a1*, and *Clcn5*. Functional annotations revealed significant alterations in subcellular localization, KEGG pathways, and GO classifications (**Supplementary Figures S2E, S3D**).

#### 3.7.2 GSEA highlights Metrnl’s modulatory effects on osteoblast and osteoclast pathways

GSEA analysis demonstrated a reduction in the Positive Regulation of Osteoblast Differentiation pathway (**Supplementary Figures S4A, B**, p-value = 0.022), suggesting that Metrnl may temper osteogenic progression. Conversely, enrichment of the Mineral Absorption pathway (**Supplementary Figures S4C, D**, p-value = 0.002) highlighted upregulated DEPs involved in calcium and phosphorus transport. Notably, a slight increase in Osteoclast Differentiation pathway proteins (**Supplementary Figures S4E, F**, p-value = 0.095) was observed but this change lacked statistical significance. This indicates a minor increase in proteins involved in osteoclast differentiation, but insufficient to significantly affect the overall process.

#### 3.7.3 Clustering analysis reveals Metrnl’s impact on osteoclast differentiation pathway

**Figure 6:**
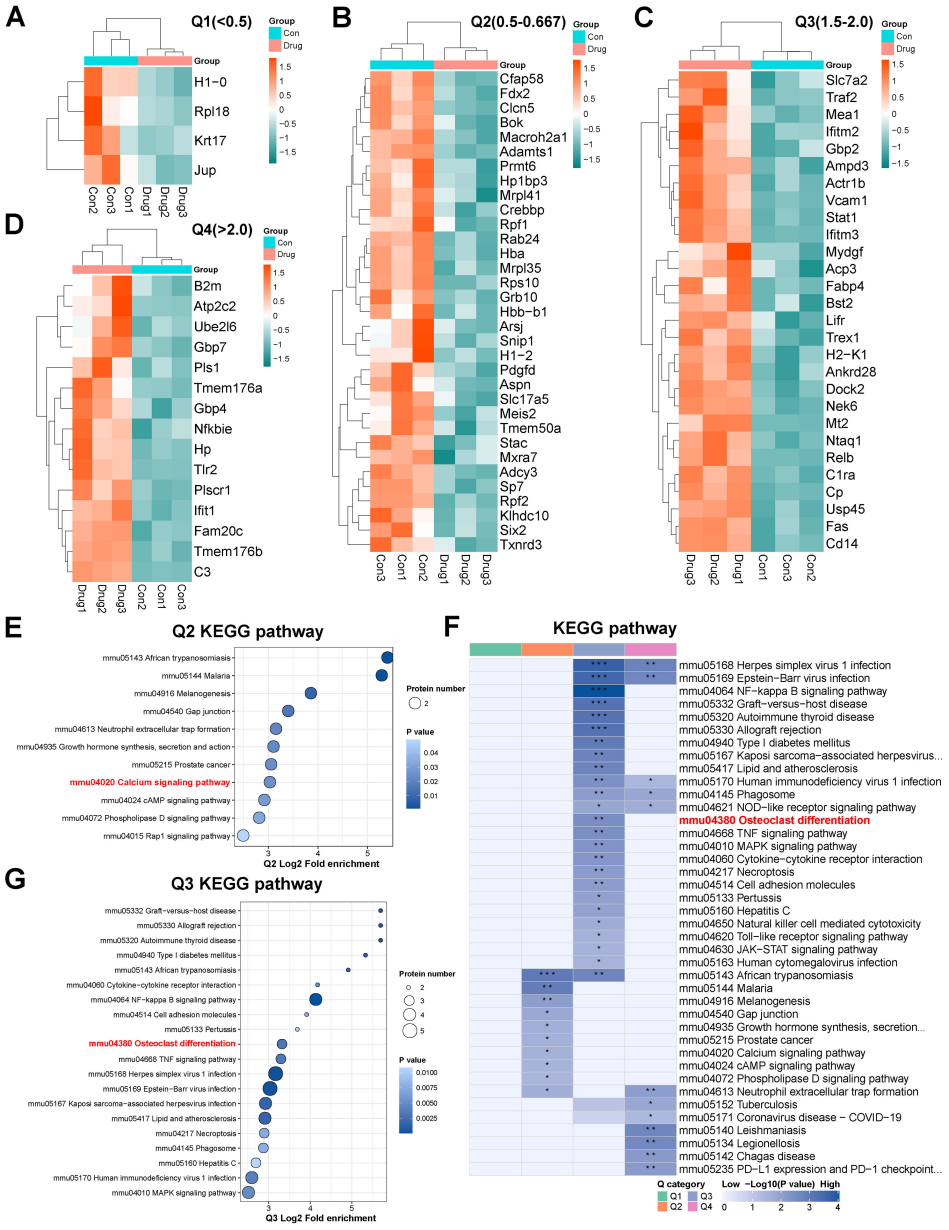
Proteomics analysis of Metrnl stimulation on MCETE-E1 cells osteogenic differentiation. **(A–D)** Q1-Q4 categories of different fold enrichment DEPs. **(A)** Heatmap of Q1 category down-regulated DEPs fold enrichment < 0.5 cluster. **(B)** Heatmap of Q2 category down-regulated DEPs 0.5 < fold enrichment < 0.667 cluster. **(C)** Heatmap of Q3 category up-regulated DEPs 1.5 < fold enrichment < 2.0 cluster. **(D)** Heatmap of Q4 category up-regulated DEPs fold enrichment > 2.0 cluster. **(E)** KEGG pathway enrichment map of Q2 cluster category DEPs. **(F)** KEGG pathway enrichment map of DEPs. **(G)** KEGG pathway enrichment map of Q3 cluster category DEPs. *p < 0.05, **p < 0.01, ***p < 0.001.

DEPs were categorized into four clusters (Q1–Q4) based on their fold enrichment values. Functional clustering analysis was performed, focusing on pathway proteins related to bone metabolism (**Figures 6A–D**). Q2 cluster analysis revealed Metrnl-mediated down-regulation of Adcy3 and Pdgfd in the Calcium Signaling pathway (**Figure 6E**), which implicates alterations in calcium ion dynamics. Furthermore, Q3 cluster analysis showed up-regulated of *Traf2*, *Stat1*, and *Relb* in the Osteoclast differentiation pathway (**Figures 6F, G**), suggesting that Metrnl may paradoxically enhance osteoclast activity during osteogenic induction.

### 3.8 Integrated multi-omics identifies Aspn and Sp7 as key targets in bone metabolism

**Figure 7:**
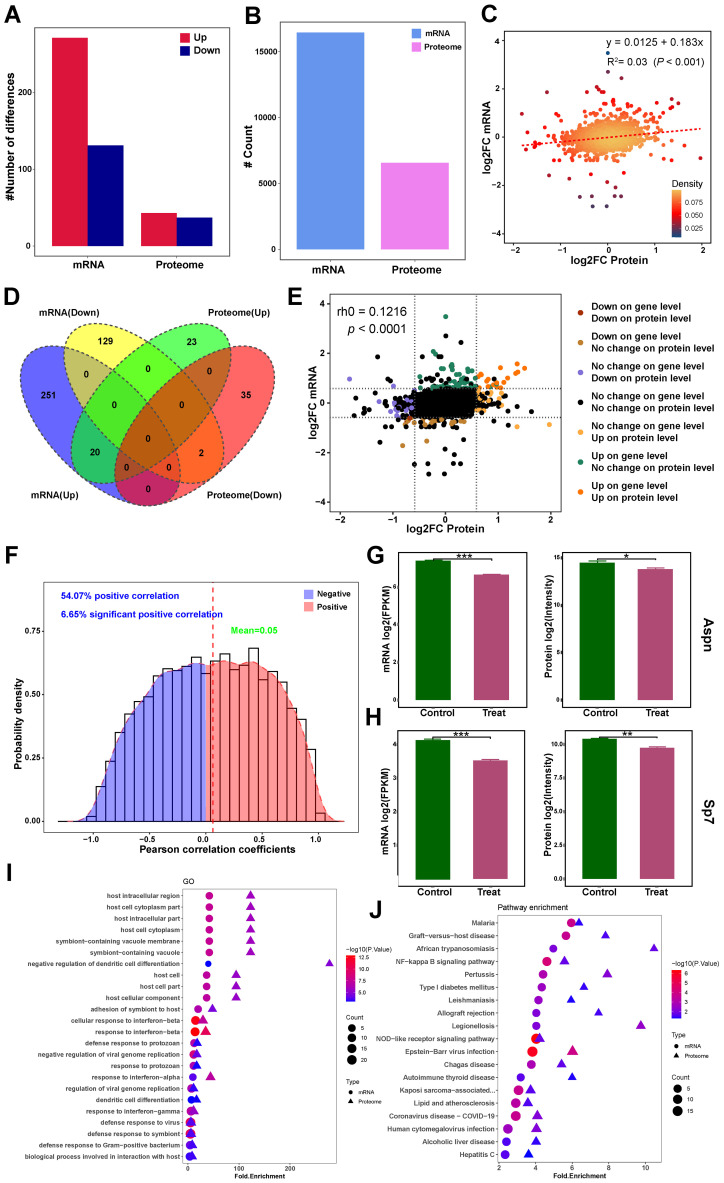
Integrated transcriptomic and proteomics analysis of Metrnl stimulation on osteogenic differentiation. **(A–F)** Correlation analysis of transcriptomic and proteomics data. **(A)** Bar plot of DEGs and DEPs number distribution. **(B)** Counts plot of mRNA and protein. **(C)** Scatter plot of correlation between transcriptome and proteome data. **(D)** Venn diagram of mRNA and proteome integrated data. **(E)** Nine-quadrant chart for correlation analysis of multi-omics integrated analysis. **(F)** Pearson correlation analyses within genes in multi-omics data. **(G)** Aspn expression in mRNA and protein levels. **(H)** Sp7 expression in mRNA and protein levels. **(I)** GO enrichment association analysis of multi-omics. **(J)** KEGG pathway enrichment association analysis of multi-omics. *p < 0.05, **p < 0.01, ***p < 0.001.

Transcriptomic and proteomic datasets were integrated to assess expression concordance (**Figures 7A, B**). Of these, 6,332 showed protein-level correspondence, while 10,118 lacked detectable protein expression. Proteomic analysis identified 6,565 genes, including 233 proteins without transcript-level matches. Cross-omics correlation analysis of these datasets revealed a weak but significant association between mRNA and protein fold changes (R²=0.03, *p* < 0.0001; **Figure 7C**). Further analysis involved creating a Venn diagram (**Figure 7D**), a nine-quadrant diagram (**Figure 7E**), and Pearson correlation analyses within genes (**Figure 7F**) to explore the intersection of DEPs and DEGs. Intersection analysis identified 22 concordant DEGs/DEPs: 20 upregulated (*Ampd3, C3, Cp, Fabp4, Fam20c, Fas, Gbp2, Gbp7, H2-K1, Hp, Ifit1, Lifr, Nfkbie, Plscr1, Relb, Slc7a2, Tlr, Tmem176, Tmem176b*, and *Vcam*) and 2 downregulated (*Aspn* and *Sp7*) (**Figures 7G, H**). Notably, the only two down-regulated DEGs/DEPs were both critical regulators of skeletal development. These findings suggest Metrnl may suppress Aspn and Sp7 to modulate bone metabolism. Enrichment analyses further supported pathway-level correlations (**Figures 7I, J**).

## 4 Discussion

In this study, we demonstrated the potential of Metrnl as a biomarker for disease activity in AS and explored its regulatory role in bone metabolism. Clinical investigations revealed that elevated serum Metrnl levels were directly correlated with disease activity in AS-Active patients, while no significant changes were observed in AS-Stable patients. Transcriptomic and proteomic analyses further elucidated Metrnl’s regulatory mechanisms, showing its inhibitory effects on endochondral ossification and its promotion of an osteoclast-conducive microenvironment, without affecting intramembranous ossification. Additionally, integrated multi-omics analyses identified key interacting genes and potential functional pathways at both the gene and protein levels.

Our clinical analysis showed significantly higher Metrnl levels in AS-Active and PsA patients compared to healthy controls (HC), but no notable differences in AS-Stable patients. Correlation analyses indicated that Metrnl levels positively correlated with inflammatory immune markers (ESR, CRP, IL-6), suggesting its association with disease activity. ROC curve analysis confirmed Metrnl’s potential as an auxiliary diagnostic marker for monitoring high disease activity in AS, although its predictive efficacy was inferior to traditional markers like ESR and CRP. The combination of Metrnl with classical indicators (C3, C4, IgG, IgA, and IgM) improved predictive accuracy, highlighting its complementary diagnostic value.

Integrated multi-omics analyses revealed Metrnl’s dual regulatory roles in osteogenesis and osteoclastogenesis. Given Metrnl’s close relationship with bone development, MC3T3-E1 cells were utilized to explore its potential connection to osteogenesis *in vitro*. While Metrnl minimally impacted intramembranous ossification (as evidenced by unaltered ALP/ARS staining), it markedly suppressed endochondral ossification through down-regulating of cartilage-associated pathways. Endochondral and intramembranous ossification represent two distinct osteogenic pathways. The former requires chondrocyte-mediated cartilage template formation prior to bone replacement, whereas the latter involves direct osteoblast differentiation from precursors (35). The experimental model employed mouse parietal bones-derived osteogenic precursor cells—a site predominantly undergoing intramembranous ossification—thereby explaining the unaltered ALP/ARS staining results. Crucially, Sp7—a master osteogenic transcription factor downstream of Runx2—was significantly inhibited at both mRNA and protein levels, suggesting that Metrnl disrupts osteoblast differentiation via Sp7 suppression (36, 37). These findings align with prior reports linking Metrnl to impaired osteoblast maturation and matrix mineralization (29).

Proteomic clustering analysis further revealed that Metrnl suppresses proteins involved in calcium signaling pathways, particularly Adcy3 and Pdgfd. Adcy3, an adenylyl cyclase, regulates cAMP levels, which subsequently influence bone cell growth and calcium channel activity (38). Pdgfd, a growth factor, promotes osteoblast and osteoclast proliferation and differentiation, playing a role in bone healing and resorption (39). Through inhibition of these proteins, Metrnl may indirectly modulate calcium signaling and bone remodeling processes. GSEA enrichment and proteomic clustering analyses indicated that Metrnl promotes genes and proteins associated with osteoclast differentiation, potentially establishing a microenvironment conducive to bone resorption. Proteomic analysis identified three enriched proteins—RelB, Traf2, and Stat1—that are involved in NFκB and RANKL pathways, critical for osteoclast activation. However, the direct effects of Metrnl on osteoclast differentiation remain unclear and warrant further investigation.

Notably, this study highlights Metrnl’s potential role in cartilage development and differentiation, which is particularly relevant to OA. Cartilage damage is a hallmark of OA, and healthy chondrocytes are essential for repair and regeneration. Recent studies have identified METRNL+ chondrocytes as a novel subtype in single-cell atlases of both normal (40–43) and OA knee cartilage, providing valuable insights into early chondrocyte dedifferentiation (44). Additionally, Metrnl has been shown to exert anti-inflammatory effects in IL-1β-induced OA chondrocytes via the PI3K/Akt/NF-κB pathway and to reduce pyroptosis through inhibition of the NLRP3/caspase-1/GSDMD pathway (16). These findings underscore Metrnl’s therapeutic potential in cartilage-related diseases.

AS is characterized by chronic inflammation and aberrant bone metabolism, with lipid metabolic dysregulation and inflammatory responses playing critical roles in its pathogenesis. Lipids within the inflammatory microenvironment act as both pro-inflammatory signaling molecules and modulators of bone cell function, contributing to the coexistence of bone destruction and pathological new bone formation in AS (45). Considering that Metrnl functions as an adipokine, a recent study that attracted our attention demonstrated that Metrnl activates the PPARα-CPT1A pathway to enhance fatty acid oxidation, thereby inhibiting abnormal lipid accumulation in nucleus pulposus cells, which reduces inflammation and attenuates intervertebral disc degeneration (46). Complementing these findings, our multi-omics analyses suggest that beyond sustaining metabolic homeostasis, Metrnl directly regulates osteogenic differentiation. Another study also confirmed that in a pro-inflammatory bone defect environment characterized by osteoblast deficiency, the targeted delivery of METRNL via a novel hydrogel (RRG-MRL) facilitates rapid restoration of post-injury microenvironmental homeostasis and enhances bone regeneration (47). By improving lipid metabolism, Metrnl may help restore homeostasis in the skeletal microenvironment, thereby reducing lipid-induced inflammatory signaling that contributes to the pro-inflammatory milieu driving pathological bone remodeling in AS. Another study also Given that abnormal bone fusion is a hallmark of late-stage AS, it is plausible that Metrnl similarly modulates lipid metabolism in immune and bone cells, mitigating inflammation and cellular dysfunction caused by lipid metabolic disturbances. Supporting this, our clinical data indicate that serum Metrnl levels are significantly elevated in active AS patients and positively correlate with inflammatory markers such as CRP, ESR, and IL-6, implying that Metrnl acts as an endogenous protective factor regulating inflammation and bone metabolic imbalance in AS. Collectively, these findings offer novel mechanistic insights into the role of Metrnl in AS. Future studies are warranted to further explore the potent regulatory mechanisms of Metrnl on bone metabolism, which may advance its development as a promising biomarker and therapeutic target for AS management. Several limitations of the present study warrant acknowledgment. Firstly, the limited sample size constrained the analysis of age- and sex-related variations, which are particularly pertinent given the well-documented sexual dimorphism in AS susceptibility. Secondly, we did not thoroughly investigate Metrnl’s association with extra-articular manifestations, such as uveitis. Moreover, the absence of spinal ligament tissue specimens precluded comprehensive analysis of local Metrnl expression at sites of injury. Bone metabolism markers and imaging data were also not systematically integrated, limiting insights into Metrnl’s role in radiographic progression. Consequently, additional systematic and comprehensive investigations are needed to elucidate the potential mechanisms of Metrnl in inflammatory immune diseases and validate its ability as a predictor for disease activity in AS.

In the second part of our study, we found that Metrnl inhibits endochondral ossification but does not appear to directly affect chondrogenesis or chondrocyte development. Proteomic analysis further indicated that Metrnl enhances the expression of proteins related to bone resorption, although experimental validation of its effects on osteoclast differentiation is still lacking. Furthermore, while multi-omics analysis indicated that Metrnl suppresses osteogenic differentiation, differences in concentration levels between *in vitro* and *in vivo* experiments raise the possibility of dose-dependent effects—such as inhibition at high concentrations and promotion at low concentrations. Further research is needed to clarify the biological roles of Metrnl in osteogenesis, osteoclast activity, and chondrocyte function across different doses, as well as to evaluate its impact on animal models of bone-related diseases.

## 5 Conclusions

In conclusion, this study evaluated Metrnl’s potential as a biomarker for AS progression and explored its role in bone metabolism using clinical and molecular approaches. Serum Metrnl levels were significantly elevated during active AS phases, correlating with disease severity, suggesting its utility as a diagnostic marker and indicator of high disease activity. Metrnl appeared to influence bone mineralization and calcification by modulating gene and protein expression, while inhibiting endochondral ossification, delaying cartilage development, and promoting osteoclast differentiation. Multi-omics analysis further revealed that Metrnl may regulate bone remodeling by suppressing Sp7 and Aspn. These observations demonstrate Metrnl’s role in maintaining bone homeostasis and its potential as a novel biomarker for AS, offering new insights into its pathogenesis and therapeutic targeting.

## Data Availability

The original contributions presented in the study are publicly available. This data can be found here: https://www.ncbi.nlm.nih.gov/bioproject?term=PRJNA1292441&cmd=DetailsSearch.
