# Supplementary material for: Metrnl/Meteorin-like/IL-41, a novel regulator of bone metabolism and disease activity in ankylosing spondylitis: based on multi-omics analysis
Source: Front Immunol. 2025 Jul 23;16:1595181. doi: 10.3389/fimmu.2025.1595181 (PMC12325251; doi:10.3389/fimmu.2025.1595181)
Supplement: Supplementary file 1 [file DataSheet1.docx]

Supplementary Material

# Exclusion/Inclusion Criteria

**Inclusion Criteria**

1. AS patients met the 2009 Assessment of SpondyloArthritis International Society (ASAS) classification criteria for axSpA.

2. PsA patients met the 2009 Group for Research and Assessment of Psoriasis and Psoriatic Arthritis (GRAPPA) diagnostic criteria

3. Age range: 16-60 years

4. Treatment-naive newly diagnosed patients

5. Signed written informed consent

**Exclusion Criteria:**

1. Concurrent autoimmune diseases

2. Severe diseases of vital organs

3. Infectious diseases

4. Pregnant women and nursing mothers: excluded for safety concerns

5. Recent use of relevant medications: such as immunosuppressants, biological agents, etc.

6. History of major surgery

7. Unable to comply with study procedures: such as those with mental disorders, cognitive impairment, etc.

# Supplementary Tables

Table S1. Demographic and clinical characteristics of AS, PsA, and HCs.

| **Characteristic** | **AS (n=150)** | | **PsA (n=20)** | **HCs (n=106)** |
| --- | --- | --- | --- | --- |
|  | **AS-Active (n=76)** | **AS-Stable (n=74)** |  |  |
| **Age, years** | 42.17 $\pm$ 12.86 | 36.3 $\pm$ 13.71 | 42.8 $\pm$ 12.64 | 29.52 $\pm$ 7.14 |
| **Sex, male/female** | 53/23 | 51/23 | 11/9 | 74/32 |
| **HLA-B27+, %** | 78.9% | 73.0% | / | / |
| **BASDAI** | 4.59 $\pm$ 0.38 | 1.54 $\pm$ 0.69 | / | / |
| **CRP** | 26.32 $\pm$ 38.52 | 2.68 $\pm$ 1.79 | 30.97 $\pm$ 54.81 | / |
| **ESR** | 28.64 $\pm$ 20.81 | 5.79 $\pm$ 4.35 | 23.05 $\pm$ 20.95 | / |
| **IL-6** | 46.12$\pm131.38$ | 1.78 $\pm$ 1.14 | 73.97 $\pm$ 212.52 | / |
| **IL-17** | 10.07 $\pm$ 2.54 | < 10.0 | 10.37 $\pm$ 3.36 | / |
| **TNF-α** | 2.16 $\pm$ 0.29 | < 2.44 | 2.15 $\pm$ 0.27 | / |
| **IFN-γ** | 2.41 $\pm$ 1.03 | < 2.44 | < 2.44 | / |
| **IgG** | 14.63 $\pm$ 4.07 | 12.24 $\pm$ 2.69 | 14.30 $\pm$ 5.05 | / |
| **IgA** | 3.79 $\pm$ 2.15 | 2.54 $\pm$ 0.81 | 3.55 $\pm$ 2.51 | / |
| **IgM** | 1.12 $\pm$ 0.69 | 1.08 $\pm$ 0.42 | 1.31 $\pm$ 0.62 | / |
| **C3** | 1.25 $\pm$ 0.28 | 1.29 $\pm$ 0.10 | 1.44 $\pm$ 0.26 | / |
| **C4** | 0.29 $\pm$ 0.10 | 0.27 $\pm$ 0.64 | 0.32 $\pm$ 0.06 | / |
| **WBC** | 7.52 $\pm$ 2.21 | 6.81 $\pm$ 1.87 | 7.83 $\pm$ 2.71 | / |
| **LY** | 4.13 $\pm$ 17.04 | 2.14 $\pm$ 0.54 | 9.75 $\pm$ 32.54 | / |
| **RBC** | 4.73 $\pm$ 0.61 | 4.95 $\pm$ 0.57 | 4.69 $\pm$ 0.48 | / |
| **HGB** | 135.61 $\pm$ 22.33 | 147.64 $\pm$ 17.64 | 139.00 $\pm$ 18.65 | / |
| **PLT** | 279.44 $\pm$ 93.58 | 247.09 $\pm$ 62.66 | 274.06 $\pm$ 82.37 | / |
| **TG** | 1.90 $\pm$ 1.25 | 1.33 $\pm$ 1.40 | 2.61 $\pm$ 2.24 | / |
| **TC** | 4.48 $\pm$ 0.89 | 4.77 $\pm$ 0.49 | 3.83 $\pm$ 0.93 | / |
| **HDL** | 0.96 $\pm$ 0.35 | 1.50 $\pm$ 0.74 | 0.85 $\pm$ 0.36 | / |
| **LDL** | 2.80 $\pm$ 0.76 | 2.76 $\pm$ 0.71 | 2.18 $\pm$ 0.78 | / |
| **AST** | 21.87 $\pm$ 10.23 | 22.85 $\pm$ 10.66 | 21.82 $\pm$ 11.63 | / |
| **ALT** | 30.73 $\pm$ 34.14 | 30.18 $\pm$ 26.99 | 34.41 $\pm$ 42.26 | / |
| **ALP** | 97.44 $\pm$ 40.05 | 76.82 $\pm$ 23.75 | 95.59 $\pm$ 50.39 | / |
| **GGT** | 41.17 $\pm$ 43.95 | 25.55 $\pm$ 18.86 | 42.94 $\pm$ 46.58 | / |
| **Urea** | 5.10 $\pm$ 1.49 | 13.48 $\pm$ 56.76 | 5.91 $\pm$ 1.77 | / |
| **Cr** | 64.41 $\pm$ 15.67 | 63.41 $\pm$ 13.65 | 66.50 $\pm$ 24.50 | / |
| **Cys-C** | 0.95 $\pm$ 0.35 | 1.07 $\pm$ 1.85 | 1.03 $\pm$ 0.37 | / |

*BASDAI, Bath Ankylosing Spondylitis Disease Activity Index; CRP, C-reactive protein; ESR, erythrocyte sedimentation rate; IL-6, interleukin-6; IL-17, interleukin-17A; TNF-ɑ, tumor necrosis factor-ɑ; IFN-γ, interferon-γ; IgG, immunoglobin G; IgA, immunoglobin A; IgM, immunoglobin M; C3, complement 3; C4, complement 4; WBC, white blood cell count; LY, lymphocyte; RBC, red blood cell count; HGB, hemoglobin; PLT, platelet count; TG, triglyceride; TC, total cholesterol; HDL, high-density lipoprotein; LDL, low-density lipoprotein; AST, aspartate transaminase; ALT, alanine aminotransferase; ALP, alkaline phosphatase; GGT, γ-glutamyl transpeptidase; Cr, creatinine; Cys-C, cysteine-C.

Table S2. Primer sequences for the mRNA of the target genes

| **Genes** | **Forward (5’-3’)** | **Reverse (3’-5’)** |
| --- | --- | --- |
| **β-Actin** | GGCTGTATTCCCCTCCATCG | CCAGTTGGTAACAATGCCATGT |
| **ALP** | CCAACTCTTTTGTGCCAGAGA | GGCTACATTGGTGTTGAGCTTTT |
| **BSP** | CAGGGAGGCAGTGACTCTTC | AGTGTGGAAAGTGTGGCGTT |
| **OCN** | CTGACCTCACAGATCCCAAGC | TGGTCTGATAGCTCGTCACAAG |
| **OPN** | AGCAAGAAACTCTTCCAAGCAA | GTGAGATTCGTCAGATTCATCCG |
| **Runx2** | ATGCTTCATTCGCCTCACAAA | GCACTCACTGACTCGGTTGG |

Table S3. Serum levels (pg/mL) of Metrnl in AS, PsA, and HCs.

| **Participants** | **Number** | **Median (pg/mL)** | **Interquartile Range (pg/mL)** |
| --- | --- | --- | --- |
| **Control** | 106 | 223.30 | 200.69 - 258.08 |
| **PsA** | 20 | 286.59 | 219.15 - 403.61 |
| **AS** | 150 | 277.57 | 218.94 - 323.98 |
| **AS-Active** | 76 | 317.64 | 283.46 - 369.80 |
| **AS-Stable** | 74 | 228.44 | 193.59 - 273.69 |

*AS, ankylosing spondylitis; PsA, psoriatic arthritis; HCs, healthy controls; AS-Active, AS patients in active period; AS-Stable, AS patients in stable period.

# Supplementary Figures

**
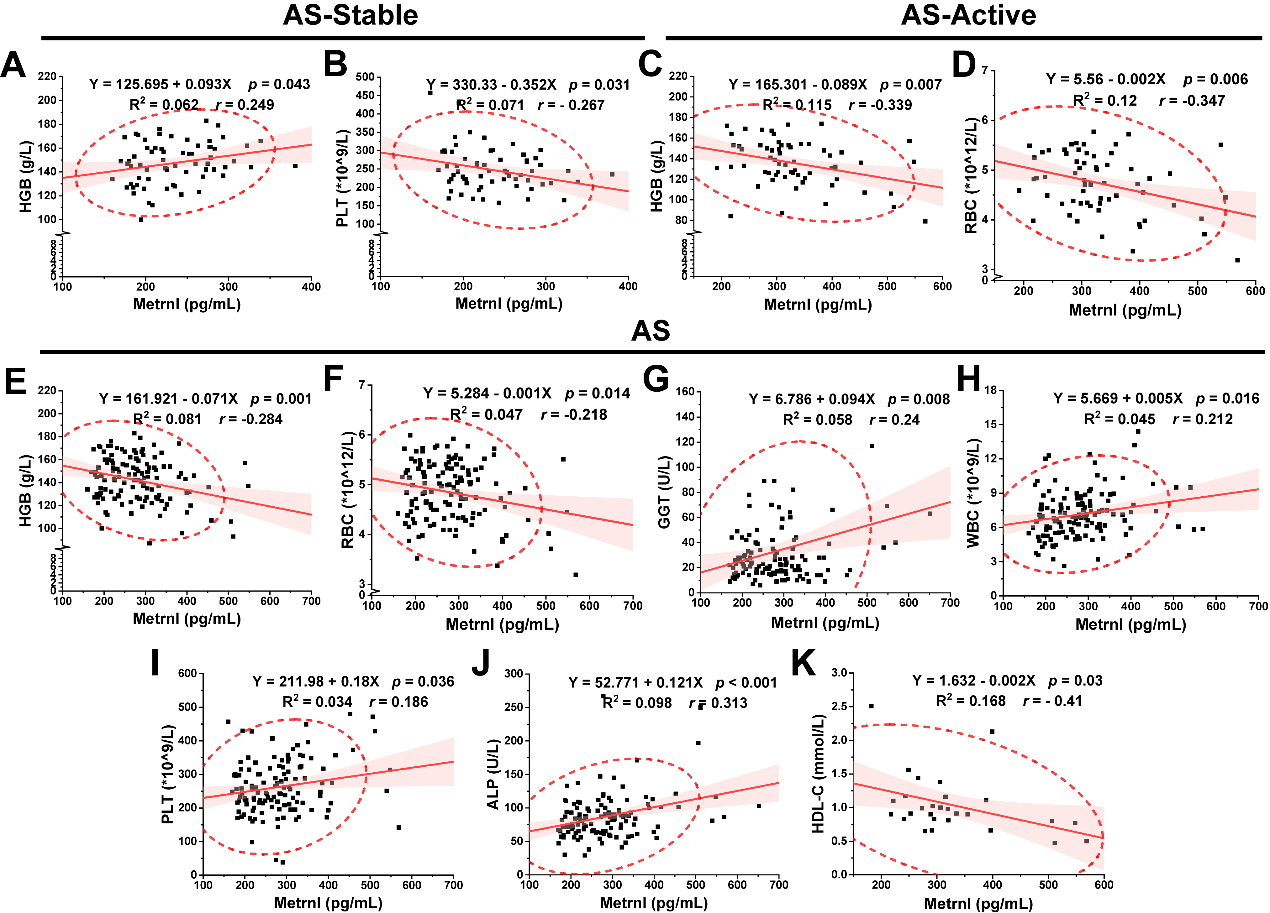
**

**Supplementary Figure 1.** Linear correlation maps between Metrnl and biochemical blood parameters. (A) Linear correlation map between Metrnl and HGB in AS-Stable period patients. (B) Linear correlation map between Metrnl and PLT in AS-Stable period patients. (C) Linear correlation map between Metrnl and HGB in AS-Active period patients. (D) Linear correlation map between Metrnl and RBC in AS-Active period patients. (E) Linear correlation map between Metrnl and HGB in AS patients. (F) Linear correlation map between Metrnl and RBC in AS patients. (G) Linear correlation map between Metrnl and GGT in AS patients. (H) Linear correlation map between Metrnl and WBC in AS patients. (I) Linear correlation map between Metrnl and PLT in AS patients. (J) Linear correlation map between Metrnl and ALP in AS patients. (K) Linear correlation map between Metrnl and HDL-C in AS patients. *p<0.05, **p<0.01, ***p<0.001.


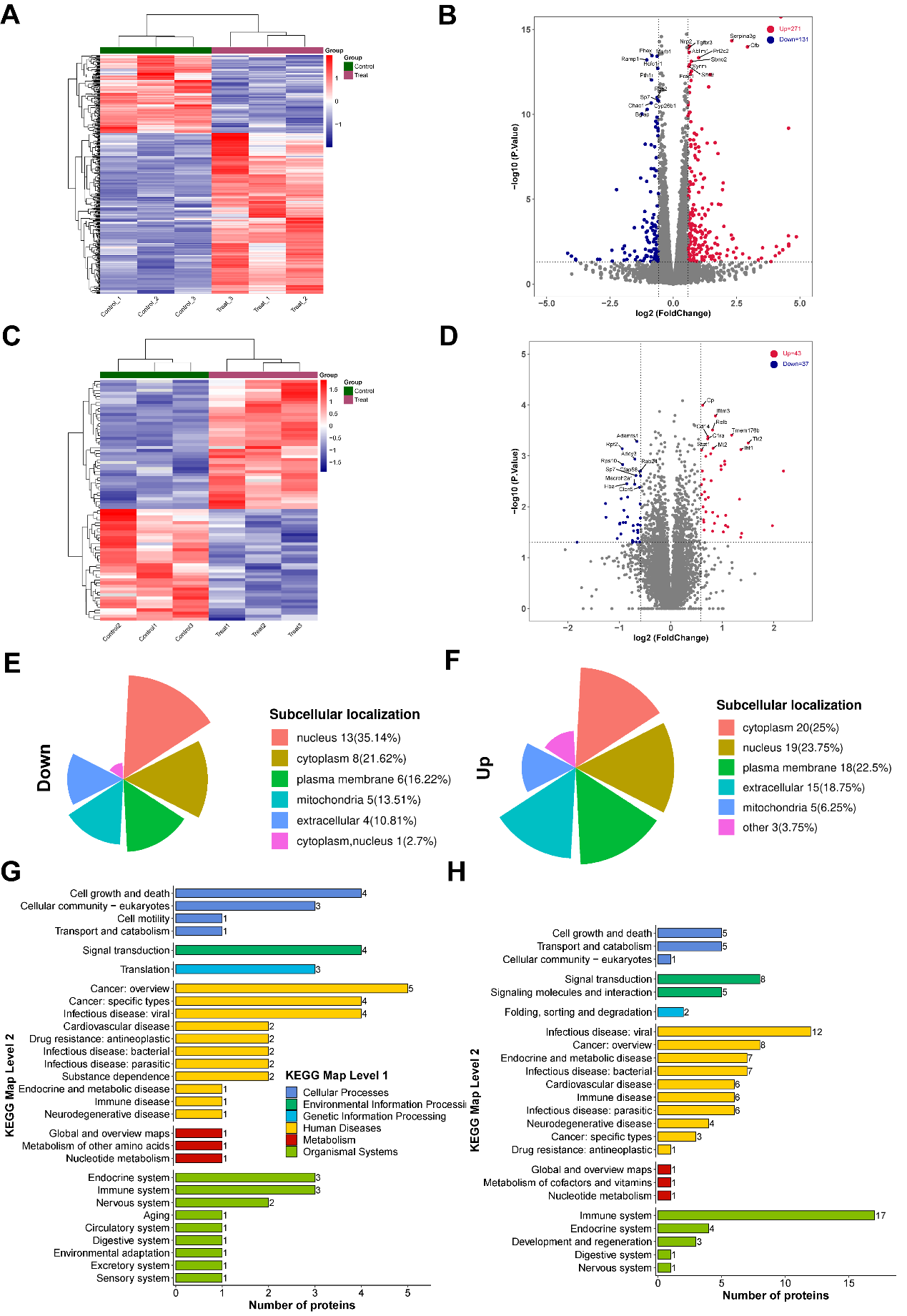


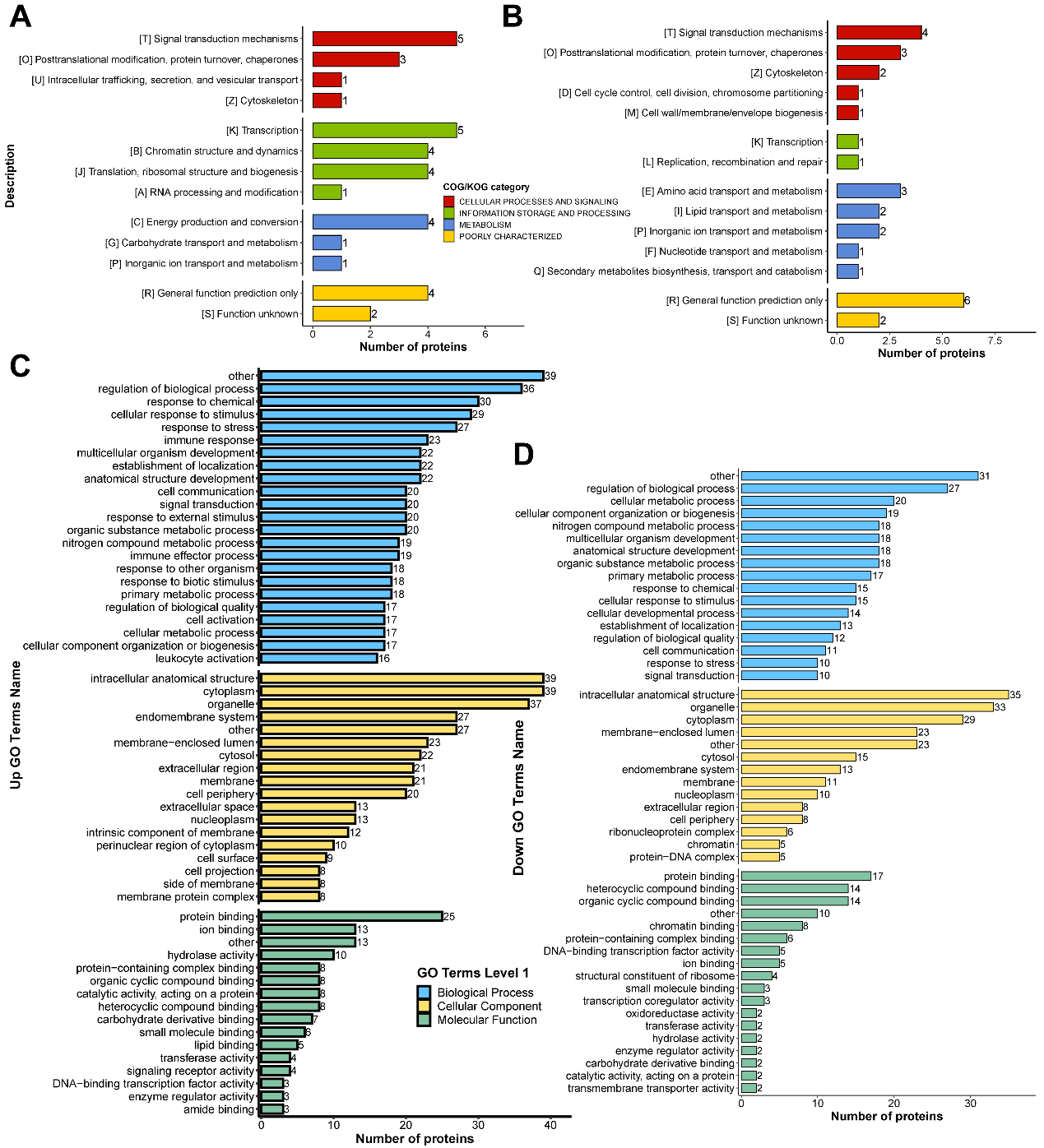
**Supplementary Figure 2.** Transcriptomic and proteomic analysis of Metrnl stimulation on MCETE-E1 cells osteogenic differentiation. (A) Heat map of transcriptomic DEGs. (B) Volcano plot of transcriptomic DEGs. (C) Heat map of proteomic DEPs. (D) Volcano plot of proteomic DEPs. (E) Down-regulated DEPs subcellular localization functional classification map. (F) Up-regulated DEPs subcellular localization functional classification map. (G) Down-regulated DEPs KEGG pathway functional classification map. (H) Up-regulated DEPs KEGG pathway functional classification map.

**Supplementary Figure** 3**.** Proteomic functional classification enrichment maps of Metrnl stimulation on MCETE-E1 cells osteogenic differentiation. (A) Up-regulated DEPs COG/KOG functional classification map. (B) Down-regulated DEPs COG/KOG functional classification map. (C) Up-regulated DEPs GO functional classification map. (D) Down-regulated DEPs GO functional
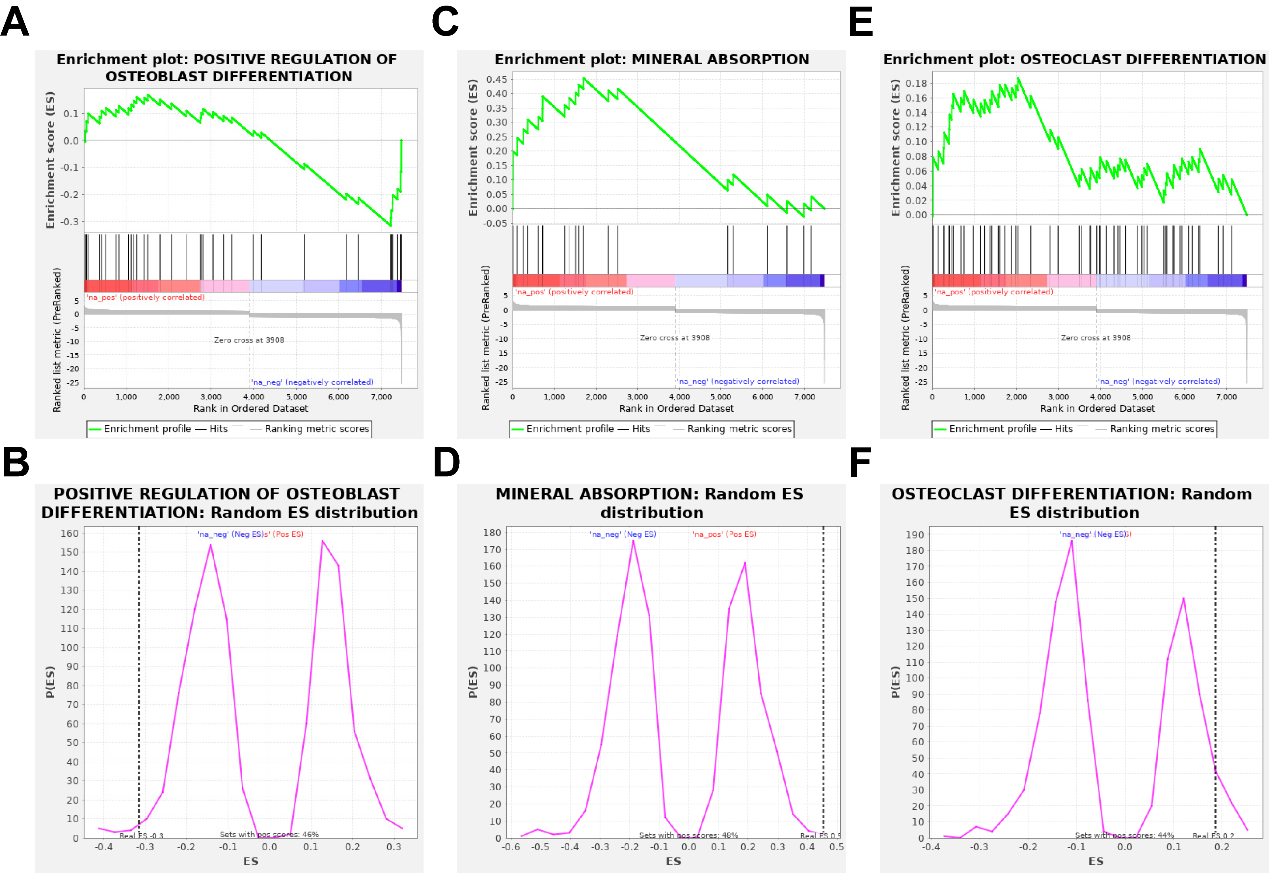
classification map.

**Supplementary Figure 4.** Proteomic GSEA analyses gene sets associated with the skeletal system. (A-B) GSEA enrichment plot of negatively regulated the Positive Regulation of Osteoblast Differentiation pathway. (A) Enrichment score (ES) profile and position of core gene sets in ranking tables. (B) Random ES distribution plot. (C-D) GSEA enrichment plot of positively regulated the Mineral Absorption pathway. (C) ES profile and position of core gene sets in ranking tables. (D) Random ES distribution plot. (E-F) GSEA enrichment plot of negatively regulated the Osteoclast Differentiation pathway. (E) ES profile and position of core gene sets in ranking tables. (F) Random ES distribution plot.
